# Editors-in-chief publishing in dental journals: Concerns in self-publishing

**DOI:** 10.1371/journal.pone.0311997

**Published:** 2024-10-11

**Authors:** Elina Nourmand, Rachel Swed, Rafael Delgado-Ruiz, Georgios Romanos

**Affiliations:** 1 Stony Brook School of Dental Medicine, Stony Brook, NY, United States of America; 2 Department of Prosthodontics and Digital Technology, Stony Brook School of Dental Medicine, Stony Brook, NY, United States of America; 3 Department of Periodontics and Endodontics, Stony Brook School of Dental Medicine, Stony Brook, NY, United States of America; Aga Khan University Hospital, PAKISTAN

## Abstract

Editors-in-chief (EICs) have a significant amount of control over the publications that are accepted in their journals, which may result in ethical predicaments. This study investigates the potential conflict of interest of EICs from various dental journals by quantifying the number of their self-published articles during their years as EIC. Based on representation across many dental disciplines and high impact factors, 67 EICs from 19 dental journals during 1990–2023 were studied. To keep anonymity, each journal was randomly assigned a letter A-S and each editor-in-chief (EIC) was given the same letter as their journal with a random number 1–67. After gathering the years each EIC served, online library resources were searched to enumerate each EIC’s lifetime publications and total self-publications during their term, excluding editorials for both counts. Descriptive statistics were performed to evaluate the results. The results indicate that 16 EICs self-published more than the average of 23.27 articles during their term. When considering EIC tenure, a ratio of self-publications per year was calculated, averaging 2.12 publications. Twenty-two EICs authored more articles annually than the average ratio. After calculating the impact factors of each EIC from the years they serviced their journal, a total of 22 impact factors exceeded the established mean of 2.45. Lastly, when comparing the percentage of self-publications from total lifetime publications, 24 of 67 EICs were above the average. Overall, a significantly increased number of self-publications was detected, presenting potential conflicts of interest for EICs. Therefore, it remains essential to develop clear guidelines and international standards regarding the practice of EICs self-publishing in their journals during their service term.

## Introduction

When a journal is inaugurated, a standard of publication ethics is established. These requirements are followed for articles before they can be published. These standards usually use a double-blind peer review process due to the bias removal from both the reviewers and authors. After passing an initial screening to determine whether a submitted manuscript is written in the appropriate format for the journal, the EIC or associate editors determine if the article can proceed to peer review or is immediately rejected. A screening process to determine the originality percentage of the manuscript content is also conducted. Generally, the criteria for immediate rejection commonly include manuscripts not within the journal scope, the information presented is not current or new, and lack of structure and order [1]. While some dental journals have a tightened process for handling editorial board members’ submitted manuscripts such as managing the peer review by alternative members of the board, most dental journals do not have an established protocol nor a readily available process for the public in this scenario [2, 3].

While some journals forbid editorial board members from publishing in their journals, others encourage their team to submit articles to their journals [4]. When editors do publish in their journals, higher odds of acceptance and quicker processing time were seen as reviewed by Scanff et al. [5]. Furthermore, Luty et al. [6] found journals are three times more likely to publish their editorial board than an outside author. However, this can be argued that the board is populated with research experts, enhancing the publishing process. Additionally, EICs aim to ensure that their journals maintain high standards of excellence, leading them to publish only their best and most impactful research. Nevertheless, this can cause a potential conflict of interest, bias, and inequity in the peer-review process [4]. Furthermore, it would be impossible to overlook the advantages that join these numerous publications such as an increase in the journal’s visibility and credibility as well as the author’s reputation and funding [2, 4]. Numerous editors have been known for a high number of self-publications in their journals while on the board [7]. Although, this can be due to the duty of an EIC to publish in their journals such as monthly editorials. Therefore, in this study editorials will be excluded to highlight publications that were approved regardless of obligations.

To evaluate the importance of a journal in their specific discipline without biases, impact factors (IFs) are used [8, 9]. As no fixed scale has been identified to classify impact factors, the best comparison is through comparing other IFs in the same field. The more review articles a journal publishes, the greater its impact factor will be, and a higher impact factor indicates a more significant journal [9]. To calculate an impact factor, the journal must be established for three years. The numerator is the number of times articles published in the last two years were cited by indexed journals during the current year. The denominator is the total number of citable items including articles and reviews published by that journal in the last two years [9]. Journal Citation Reports is the primary resource for impact factors and has been used in this study to collect the IFs to see if a trend exists between impact factors and self-publishing editors in chief (EICs).

As the highest authority of the editorial board, an EIC is responsible for overseeing the publication’s operations and policies. Hence, an EIC is responsible for establishing the utmost ethical standards for their journal. This study aims to evaluate the self-publishing practices of EICs of dental journals during their service and to determine if potential conflicts of interest exist.

## Methods

In this study, 67 EICs serving between the years 1990–2023 in 19 dental journals with high impact factors from various dental specialties were analyzed. The 19 journals were selected randomly to represent more than 20% of dental journals with impact factors. Each journal’s “Guide for authors” was searched for guidelines and policies pertaining to the self-publications of editorial board members. To keep anonymity of the journals and EICs, each journal was randomly assigned a letter A-S, and each EIC was given the same letter as their journal with a randomly assigned number 1–67. IRB approval and individual consent were not required as data was analyzed anonymously without intervention or interaction with the individuals; additionally, identifiable private information was not analyzed nor obtained. Descriptive statistics were performed to present the mean values of articles published during each EIC’s service period, the corresponding impact factors, the ratio of self-publications while EIC per year of service, and the percentage of self-publications in relation to their total lifetime publications.

After gathering the years each EIC served in their role, the online database of each journal was noted. Once on each journal’s online website through the database, each EIC was searched to enumerate the total articles authored in their journal during their career with the search query of [Last name, First name] as well as the filters of “Author” and the specific range of years in their role. If the author had a middle name, the initial was included after the first name in the query. This count was subtracted from the number of editorials published since they are solely published due to their role as EIC, so it would sway the counts of their publications. If the online resource of a journal did not have an efficient search engine, then a manual search was conducted by sifting through each article’s authors during the years of each EIC while verifying editorials were still excluded. To count the total articles published in each EIC’s lifetime, PubMed, Web of Science, and ResearchGate were searched either by viewing the profile of the EIC or by using the same search query mentioned above without the limitation of years.

Editorials were excluded in this count too. All articles studied were either authored by the EIC in isolation or co-authored.

Lastly, the yearly impact factor of each journal was collected from Journal Citation Reports’ individual yearly impact factors. Then, using the impact factors only during the years of each EIC’s service, a mean was calculated for each EIC which is referenced in this study as “average impact factor.” In essence, the “average impact factor” is the journal’s IF during the years of each EIC studied. However, impact factors for all years were not reported, so those authors who were EIC during those missing years were excluded from these results. Additionally, impact factors for years 2022 and 2023 have not yet been released, so the most recent impact factors from 2021 were used. This information was used to see if a correlation or causation can be made between impact factors and publications.

## Results

After analyzing the policies of author publications, it was found that all 19 dental journals lacked guidelines pertaining to the self-publications of editorial board members. Therefore, a complete study was conducted to view the results of the publishing habits of the 67 EICs.

**Fig 1:**
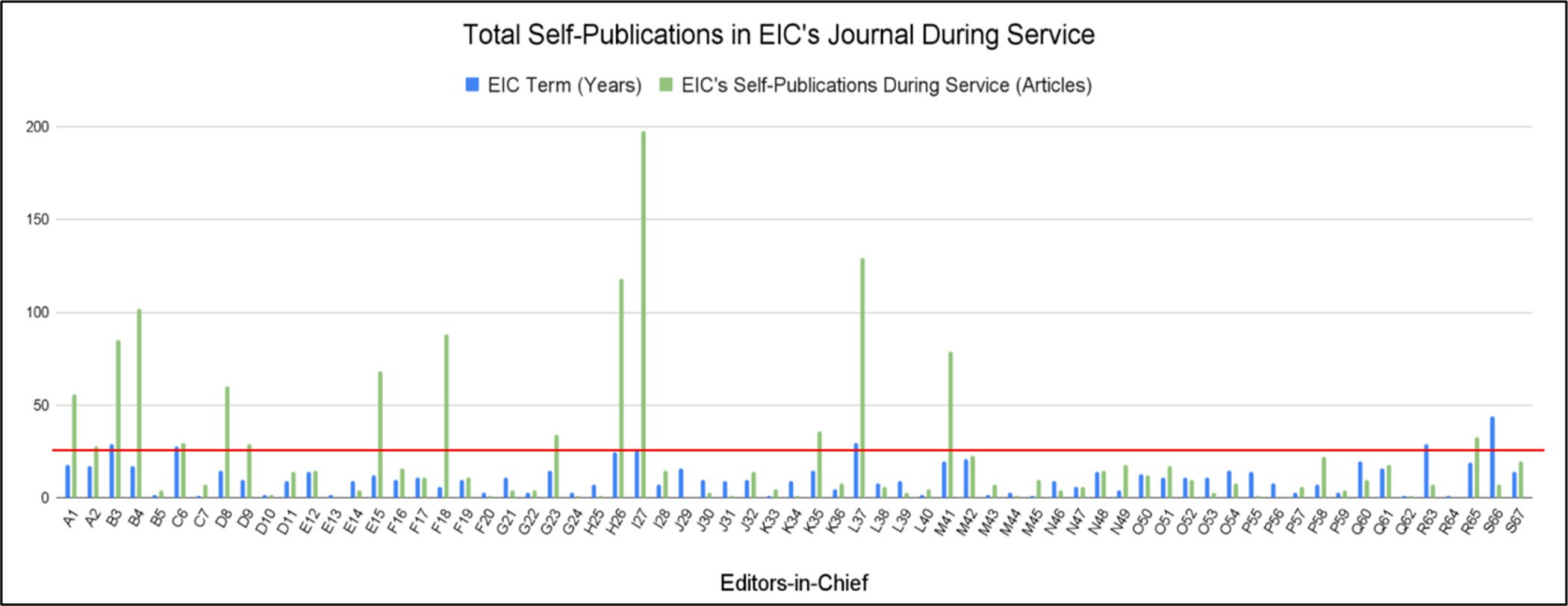
Total self-publications in EIC’s journal during service. Fig 1 compares the years each editor served as EIC (blue bars) with the number of publications in their journal during their years as EIC (green bars). An average of the articles published by all the EICs during their service in their journal is depicted in the red horizontal line. For instance, I27 served as EIC for 26 years and surpassed the average number of articles by publishing 198 articles in Journal I during those years of service.

Firstly, as seen in Fig 1, a comparison was done to plot the number of years each EIC served in their role (blue bars) with the number of articles each EIC published in their journal during their term (green bars). The average number of articles published among the EICs studied was found to be 23.27 articles as indicated by the red horizontal line. While most EIC published below the average article count, 16 editors exceeded the average with some editors publishing in the hundreds. However, since each EIC served a different number of years, this average cannot accurately depict the ethics of each EIC. Instead, the number of articles published can be justified for some EICs when the years of service as an EIC are also considered. When viewing the blue bars and green bars side-by-side, it becomes evident that 17 editors published substantially more than the number of years as EIC: A1, A2, B3, B4, D8, D9, E15, F18, G23, H26, I27, K35, L37, M41, N49, P58, and R65.

**Fig 2:**
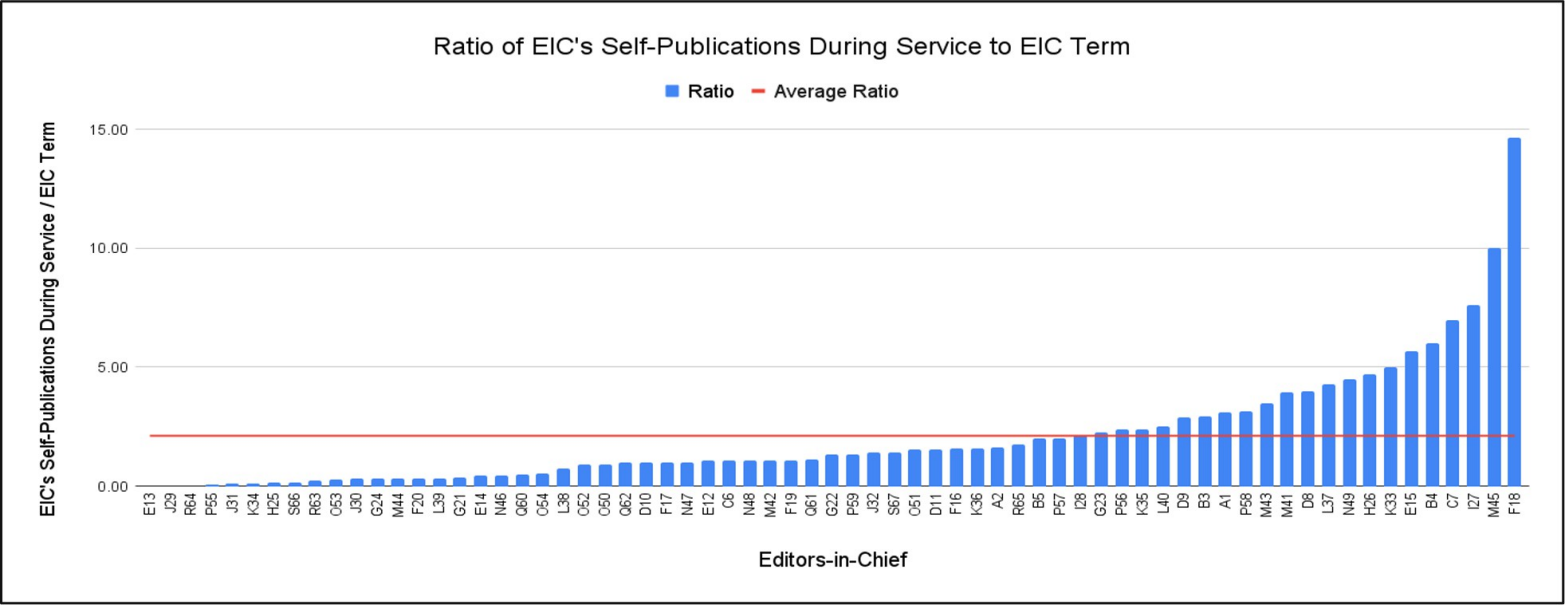
Ratio of EIC’s self-publications during service to EIC term. Fig 2 graphs the ratio of each EIC’s publications in their journal while serving as EIC over the total years served as EIC. The red horizontal line depicts an average between all the ratios calculated for the 19 EICs. For instance, F18 published 88 articles in Journal F over six years of serving as EIC; therefore, F18 exceeded the average ratio by publishing ~14.67 articles per year while EIC in their journal.

To account for both the number of years as EIC as well as the number of publications in their journal during that time, the ratio for each EIC was calculated and plotted in Fig 2. The average ratio was found to be 2.12 articles per year as depicted by the red horizontal line with a standard deviation of 2.55. Therefore, 67.2% of authors published below this average ratio, while 22 EICs were found to author more articles than the average ratio: I28, G23, P56, K35, L40, D9, B3, A1, P58, M43, M41, D8, L37, N49, H26, K33, E15, B4, C7, I27, M45, and F18.

**Fig 3:**
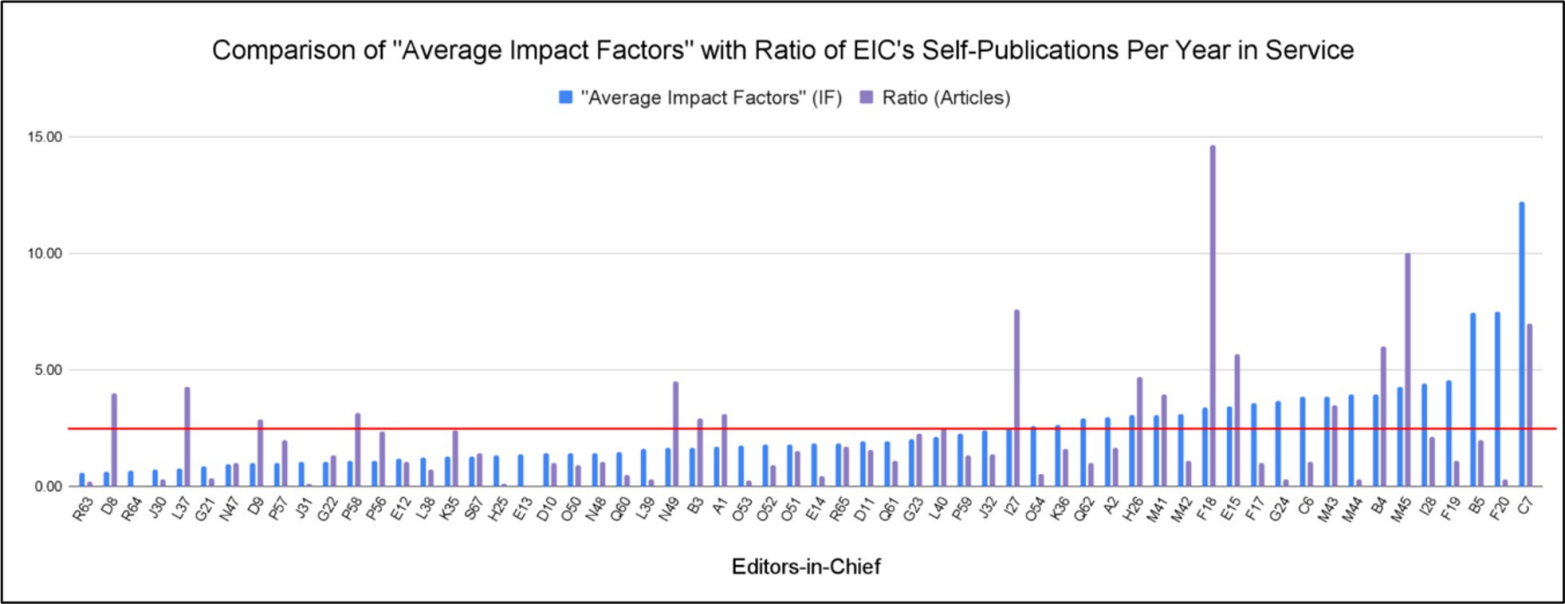
Comparison of “average impact factors” with ratio of EIC’s self-publications per year in service. Fig 3 illustrates the “average impact factor” over the years each EIC served their term in their journal (blue bars) and the ratio of publications in each journal while EIC over the years as EIC (purple bars). The red horizontal line depicts the mean of the “average impact factors” between the EICs during their years of service in each journal. For instance, the “average impact factor” of F18 is calculated by averaging the yearly impact factors of Journal F during F18’s years as EIC; this was found to be 3.38 IF and can conveniently be compared to F18’s ratio established in Fig 2 with the adjacent bars placed here.

As impact factors do not possess a predefined baseline for comparison but are instead evaluated in relation to other journals within the same field, we have computed a mean of the “average impact factors” of 2.45 based on the journals and years examined in this study. This value is represented by the red horizontal line in Fig 3. The results indicated 63.3% of the “average impact factors” were below the mean while 22 “average impact factors” were above this computed average. These “average impact factors” were compared to the ratio of publications of each EIC while serving in their journal over the years as EIC. Although the “average impact factors” were above the mean for I27, H26, M41, F18, E15, M43, B4, M45, and C7, indicating higher quality research, their ratios were still higher than the average ratio of 2.12 indicated earlier. Other EICs with higher-than-average ratios have not received prioritization in this study due to their slight justification from their low “average impact factors.”

**Fig 4:**
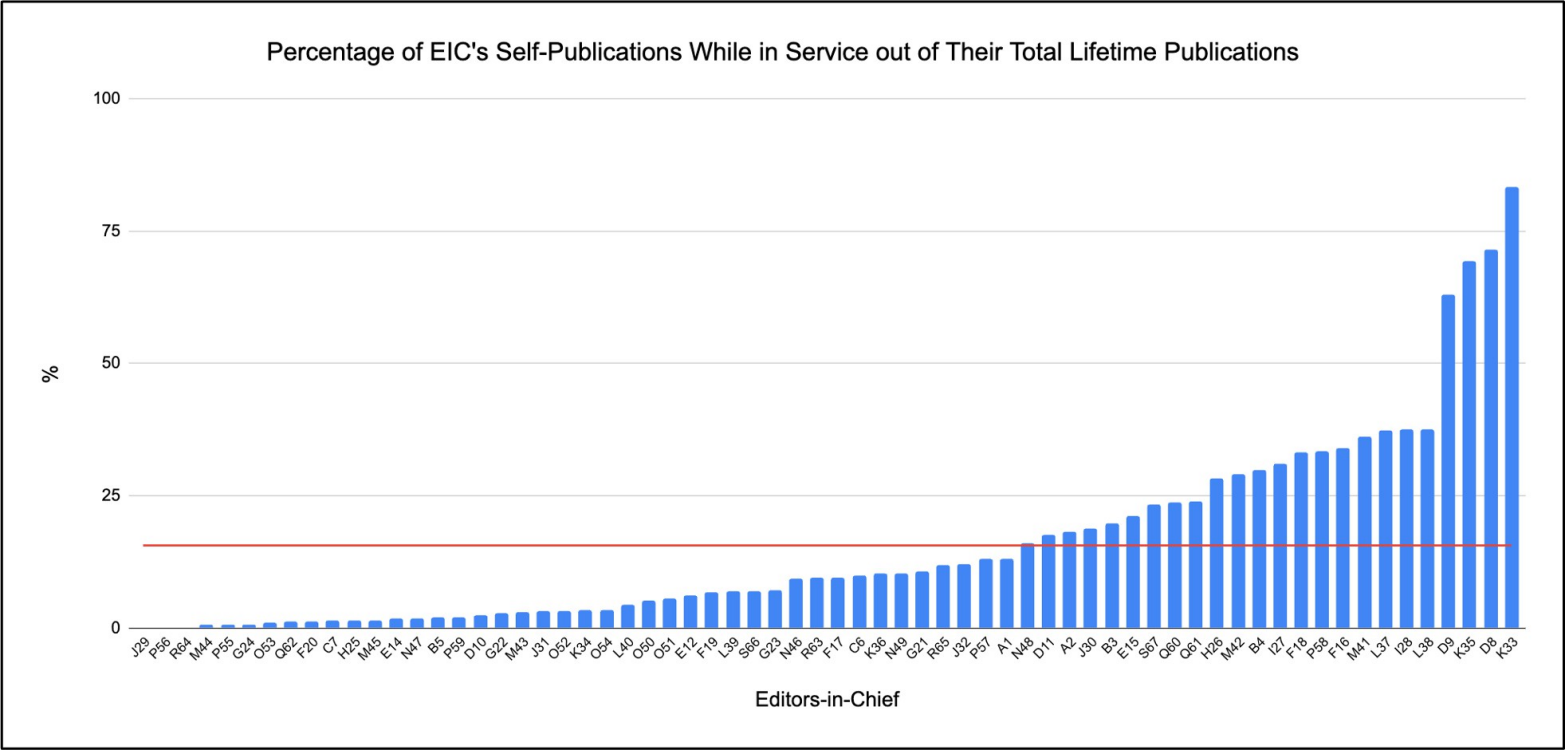
Percentage of EIC’s self-publications while in service out of their total lifetime publications. Fig 4 demonstrates the percentage each editor published during their EIC term in their journal compared to their total lifetime publications. The red horizontal line depicts an average percentage of 15.61. For instance, ~83.33% of K33’s work has been articles published in Journal K during their term as EIC, which is beyond the average percentage.

Lastly, to synthesize the results, Fig 4 depicts the percentage of published articles during each EIC’s term in their journal over their total lifetime publications. This offers a glimpse of how many of their total publications were published during their time as EIC in their journal. The average editor published 15.61% of their lifetime articles during their EIC tenure in their journal as depicted by the red horizontal line. The standard deviation was 18.42% between all the EICs. While 43 editors were below the average percentage of self-publications over total publications, 24 editors were above the average: N48, D11, A2, J30, B3, E15, S67, Q80, Q61, H26, M42, B4, I27, F18, P58, F16, M41, L37, I28, L38, D9, K35, D8, and K33.

## Discussion

It was found that 17 EICs self-published a significantly higher number of articles during their service compared to the other 50 EICs. In fact, when accounting for the ratio of their self-publications over the years they served as EIC, even more editors were found to be above the average ratio of 2.12 as seen in Fig 2. Although an EIC has the power to shape future research in their field and build connections with others, there is the disadvantage of losing time for their research [3]. Therefore, EICs might attempt to get the best of both worlds by increasing their self-publications through their editorial board role.

In Fig 3, a mean 2.45 was found when comparing the “average impact factors” of the studied EIC. Since articles self-published by EICs tend to be published speedily due to favoritism, their writings might have quality issues [4]. Lower-quality articles can lower the impact factor of a journal. Therefore, it is understandable that 63.3% of the studied “average impact factors” were below the mean due to the self-publications. On the other hand, journals with lower impact factors can be “excused” for many self-publications because of the negative assumptions that come with a journal with a low impact factor. In fact, those EICs who are self-publishing in journals with higher impact factors should be examined further due to the elevated expectations of such an impactful journal.

Overall, a significant potential for conflicts of interest was determined, as Fig 4 found that 24 of the 67 EICs published above the average of 15.61% of their lifetime articles during their EIC tenure in their journal. In other words, over of a quarter of the studied EICs self-published 15.61% of their total publications during their time as EIC.

Considering all the findings, six authors were flagged for being above average for all four results: I27, H26, M41, F18, E15, and B4. This signifies that these EICs self-published more articles than the average during their EIC term, had a higher-than-average “average impact factor,” and their percentage of self-publications out of total lifetime publications was above average. Although this can be due to doing their job correctly because ultimately, they are editors who enjoy conducting research, this can also be seen in a negative light when done without the right intentions such as honor or increasing visibility of their authorship and journal.

As with all studies, we experienced a few limitations when gathering our data. Firstly, some authors had the same name as other authors, making it difficult to tally the number of publications. We filtered by topic when we searched to mitigate this issue. Also, some journals did not have impact factors that dated back, so those impact factors were not included in this study. Additionally, certain databases would have various labeling for the same article, i.e., labeling an editorial as a review article. Therefore, we sifted through the articles that we doubted manually.

Editorial ethics is a critical yet rarely discussed subject that needs to be addressed due to the high potential for conflicts of interest. Despite being appointed based on their merits, EICs are still human, and their morals may be subject to challenges from their biases when publishing in one’s journal. Although the International Committee of Medical Journal Editors (ICJME) states in their guidelines that journals should have an outlined policy for manuscripts submitted by those involved on an editorial board, the exact recipe to these necessary guidelines are not explicitly detailed [10]. Therefore, editors can do as they wish without being held accountable. While the Committee of Publication Ethics (COPE) holds EICs should not be denied rights to publish in their own journal, they do admit it can be challenging to manage any conflicts of interest and so extra precautions should be taken. Few solutions exist but COPE’s guidelines on Editors Editing for New Editors recommends describing the process of how the peer review procedure was managed for submissions from editorial board members [11]. COPE has a forum to submit specific cases to receive insight on publication ethics, which can be useful for cases with EICs publishing in their own journal. Additionally, for greater transparency, journals can demonstrate that other associate editors can freely reject the EIC to prevent a culture that lacks integrity within the editorial board, jeopardizing the general trust in journals. Journals can join professional organizations that holds them accountable and have an outside governing board who weigh in on conflict of interests to allow the journal’s success.

## Conclusion

Considering the findings of this study, a potential conflict of interest was determined for EICs publishing in their journal during service.
